# Buffalo Cardio-Metabolic Occupational Police Stress (BCOPS) study: a seven- and twelve-year prospective analysis of occupational exposures and health outcomes among police officers

**DOI:** 10.1007/s00420-025-02142-x

**Published:** 2025-05-23

**Authors:** John M. Violanti, Desta Fekedulegn, Cecil M. Burchfiel, Erin McCanlies, Samantha K. Service, Anna Mnatsakanova, Ja K. Gu, Penelope Allison, Micheal E. Andrew, Luenda E. Charles

**Affiliations:** 1 Department of Epidemiology and Environmental Health, School of Public Health and Health Professions, University at Buffalo, The State University of New York, Buffalo, NY USA; 2 Health Effects Laboratory Division, Centers for Disease Control and Prevention, National Institute for Occupational Safety and Health, Morgantown, WV USA; 3 Department of Behavioral Medicine and Psychiatry, Faculty and Staff Assistance Program, West Virginia University, Morgantown, WV USA

**Keywords:** Police officers, Health, Cardiovascular disease, PTSD, Depression, Stress

## Abstract

**Objective:**

Overall, police officers have higher rates of several adverse health conditions (e.g., cardiovascular health profiles and post-traumatic stress disorder (PTSD)) compared to persons in many other occupations. Our objective was to conduct a comparative study of occupational exposures and health outcomes among police officers across: (a) a 7-year period, from the baseline examination (2004–2009) to the 1st follow-up examination (2011–2015) and (b) a 12-year period, from baseline to the 2nd follow-up examination (2015–2019).

**Methods:**

Participants were from the Buffalo Cardio-Metabolic Occupational Police Stress (BCOPS) Study. Variables were assessed through self-report, standardized validated questionnaires, or standardized medical procedures. We computed the 7- and 12-year changes in mean values (for continuous/numeric variables) or prevalence (for categorical variables) and the corresponding 95% confidence intervals (CIs) using MIXED and GENMOD procedures in SAS.

**Results:**

Occupational stress significantly increased over 12 years [3.4; (95% CI 1.2, 5.6)]. The percentage of officers who reported excellent/very good health significantly decreased across both time periods: [− 11.8%; (− 17.8, − 5.9)] across seven years and [− 17.3%; (− 24.2, − 10.4)] across 12 years. The prevalence of metabolic syndrome increased over seven years [10.7%; (5.3–16.0)] and over 12 years [7.4%; (0.1–14.0)]. Abdominal obesity and glucose intolerance significantly increased over both time periods while hypertension and elevated triglyceride levels increased slightly but not significantly over both time periods.

**Conclusion:**

Occupational stressors and some health outcomes of officers worsened over time indicating the need for self-health monitoring and wellness programs for police.

## Background

Overall, police officers have higher rates of several adverse health conditions compared to persons in many other occupations (Casas and Kegel 2024; Joseph et al. 2009; Ramey et al. 2009; Violanti et al. 2009). They have worse cardiovascular profiles, higher levels of traditional cardiovascular disease (CVD) risk factors such as cholesterol and metabolic syndrome as well as non-traditional risk factors such as post-traumatic stress disorder (PTSD) and depression (Andrews et al. 2023; Carleton et al. 2020; Franke, et al. 2002; Gendron et al. 2019; Greeshma et al. 2024; Hartley, et al. 2011; Violanti, et al. 1996; Zimmerman 2012). The average age for a police officer who has suffered a heart attack is 49 years old compared to 67 years of age for individuals in the general population (Kulbarsh 2009). Studies of police officers found that all-cause mortality for white male officers was significantly higher than expected (Violanti et al. 2013; Vena et al. 2014; Vena et al. 1986).

This health disparity may in part be due to the many occupational stressors to which the officers are regularly exposed (Andrews et al. 2023; Carleton et al. 2020; Casas and Kegel 2023; Joseph et al. 2009; Padilla 2020; Ramey. et al. 2009; Violanti et al. 2013). These stressors include exposure to traumatic events (e.g., motor vehicular accidents, seeing dead bodies and abused children (Ramey et al. 2009; Hartley et al. 2007; Violanti et al. 2016) and organizational stressors (e.g., long work hours, shiftwork, staff shortages, paperwork, and concern about physical harm (Violanti et al. 2016; Shane 2010). These stressors may even be greater among officers who belong to various sub-groups of the population (Kim et al. 2024; Padilla 2020; Violanti et al. 2016; He et al. 2002; Yoo et al. 2011).

Investigations of police officers’ health have shown worse health outcomes overall (Gendron et al. 2019; Greeshma et al. 2024; Franke et al. 2002; Hartley et al. 2011; Violanti et al. 2009, 1996). One study reported a somewhat positive picture (Lockie et al. 2022) than has been reported in most studies. However, the health of police officers remains a public health concern that deserves continuous attention. Therefore, we decided to undertake a comparative study. This study is unique because there are few longitudinal studies that have evaluated the health effects of occupational stress in police officers and, to our knowledge, none have over 12 years of follow-up (Hansen et al. 2022; Magnavita et al. 2018). This is particularly relevant given that many health outcomes including diabetes, metabolic syndrome, and cardiovascular disease that are being assessed as part of the longitudinal study can take years to manifest. The objective of this study was to examine and describe changes in occupational exposures and health outcomes experienced by police officers across two time periods: (a) a seven-year period from the baseline examination (2004–2009) to the 1st follow-up examination (2011–2015) and (b) a 12-year period from baseline to the 2nd follow-up examination (2015–2019). This paper is descriptive in nature; we are not examining exposure-outcome relationships. The focus is to present the magnitude of changes in occupational exposures and health outcomes across time as such data are rarely available for police officers in the scientific literature.

## Methods

### Participants and data sources

**Fig. 1:**
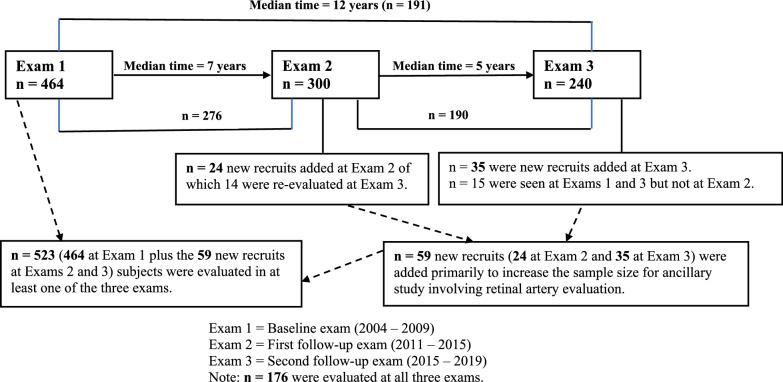
The Buffalo Cardio-Metabolic Occupational Police Stress (BCOPS) Study Design and Sample Size

The Buffalo Cardio-Metabolic Occupational Police Stress (BCOPS) Study was initiated to investigate associations between stressors unique to the law enforcement profession and psychological and physiological health outcomes. Comprehensive study details can be found elsewhere (Violanti et al. 2006). Three examinations have been completed (Fig. 1). a baseline examination with 464 officers (2004–2009), the 1st follow-up examination (300 officers; 2011–2015), and the 2nd follow-up examination (240 officers; 2015–2019). The number of officers who participated in both the baseline and 1st follow-up examinations was 276, while those who participated in both the baseline and 2nd follow-up examinations was 191. For the baseline examination, a total of 710 police officers who worked with the Buffalo, New York Police Department were invited to participate. Four hundred and sixty-four (65.4%) active-duty and retired officers agreed to participate and were examined during 2004 to 2009. No specific inclusion criteria were indicated for the study, only that the participants be sworn officers and willing to participate. Pregnant women were excluded from all examinations. All exams were conducted in the Center for Health Research, School of Public Health and Health Profession, the State University of New York at Buffalo (SUNY-Buffalo). The study was reviewed and approved by the Institutional Review Board of the SUNY at Buffalo. All participants signed a consent form.

### Demographic and lifestyle factors

Demographic characteristics (e.g., age, gender, race/ethnicity, education, marital status, years employed as a police officer, rank), lifestyle behaviors (e.g., smoking status, alcohol intake), and general health status information were obtained using self-administered questionnaires. General health status was assessed with the question “In general, would you say your health is: 1—Excellent; 2—Very good; 3—Good; 4—Fair; or 5—Poor?”.

### Occupational factors

*Work Hours*. Information on work hour variables (i.e., hours of regular time and overtime work per week, and hours worked on a second job) was collected using a self-administered questionnaire. The officers were asked the following series of questions: “On average, how many hours a week do you work at your regular shift?”, “On average, how many overtime or extended hours do you work per week at your police job?”, and “Do you work a second job (yes/no)? and If YES, how many hours a week do you work at your second job?”.

*Shiftwork*. Day-by-day electronic work history records were available for a 15-year period spanning from 1994 or first date of employment to date of each examination from which the dominant shift across their career was derived. Complete details of shiftwork assessment are provided elsewhere (Fekedulegn et al. 2013).

### Psychosocial factors

Several self-administered questionnaires were used to assess psychosocial factors among the officers (Table 1). The Perceived Stress (PSS-14) scale measured feelings about personal problems and stressful events (Cohen et al. 1983). The Center for epidemiological studies-depression scale (CES-D) measured depressive symptoms (Radloff 1977). The PTSD Checklist-Civilian version (PCL-C) assessed PTSD symptoms (Weathers. et al. 2013, https://ptsd.va.gov/professional/assessment/adult-sr/ptsd-checklist.asp; updated on 10/20/2024). The Beck Anxiety Inventory (BAI) measured the emotional, physiological, and cognitive symptoms of anxiety (Beck et al. 1988). The Beck Hopelessness Scale (Beck et al. 1985) assessed the degree of negative expectations about the future. The Cook-Medley Hostility Scale (Cook 1954; Barefoot et al. 1989) measured personality and temperament. The PTSD Checklist -civilian version (PCL-C) (Weathers et al. 2024) measured symptoms of posttraumatic stress. The Spielberger Police Stress Survey (Martelli et al. 1989; Spielberger et al. 1981) measured exposure to potentially stressful events or conditions relevant to police work. The Brief.

**Table 1:** Characteristics of selected instruments of psychosocial and other measures used in the BCOPS study

Subject	Instrument	Description	Number of items	Score calculation	Subparts	Score range	Interpretation of higher score
Perceived stress	Perceived stress (PSS-14) scale	Feelings about personal problems and stressful events over the past month	14 items on a 5-point scale ranging from 0 to 4	Sum of all items, after reverse coding selected items	na	0–48	Worse
Depressive symptoms	Center for epidemiological studies-depression scale (CES-D)	Depressive symptoms over past week	20 items on a 4-point scale ranging from 0 to 3	Sum of all items, after reverse coding selected items	na	0–60	Worse
PTSD symptoms	PTSD checklist-civilian version (PCL-C)	How bothersome certain stressful life experiences were in past month	17 items on a 5-point scale ranging from 1 to 5	Sum all items	Clusters: re-experiencing, avoidance/numbing, arousal	17–85	Worse
Anxiety	Beck anxiety inventory (BAI)	Emotional, physiological, and cognitive symptoms in past week	21 items on a 4-point scale ranging from 0 to 3	Sum all items	na	0–63	Worse
Hopelessness	Beck hopelessness scale	Degree of negative expectations about future	20 items true–false	Sum of all items, after reverse coding selected items	na	0–20	Worse
Hostility	Cook-medley hostility scale	Personality and temperament, degrees of hostility	50 items true–false	Sum of select items for each subscale	Subscales: cynicism, hostile attributions, hostile affect, aggressive responding, social avoidance, other	Varies by subscale	Worse
Posttraumatic stress	Impact of events-revised (IES-R)	Subjective impact or symptoms related to a traumatic event	22 items on a 5-point scale ranging from 0 to 4	Sum all items	Subscales: intrusion, avoidance, hyperarousal	0–88	Worse
Police stress	Spielberger police stress survey	Number and intensity of events (range: 1–100) in the past month and year	60 items	Sum of intensity	Subscales: admin./professional pressure, physical/psychological danger, lack of support	Varies by subscale	Worse
Coping	Brief COPE	How officers cope with stress	28 items on a 4-point scale ranging from 0 to 3	Average score of the items in each factor	Factors: active coping, passive coping, support seeking*	Varies by factor	Active coping and support seeking = better Passive = worse
Hardiness	Hardiness scale	Personality trait that may influence one’s perception of stressful circumstances	15 items on a 4-point scale ranging from 0 to 3	Sum of all items, after reverse coding selected items	control, commitment, and challenge	0–45	Worse
Social support	Social provisions scale	Social support from family members, co-workers and community members	24 items on a 4-point scale ranging from 1 to 4	Sum of all items, after reverse coding selected items	Provisions: guidance, reliable alliance, attachment, social integration, reassurance of worth, opportunity for nurturance	24–96	Better
Sleep	Pittsburgh sleep quality index (PSQI)	Sleep quantity and quality over past month	19 items; 4 in hours, others on a4-point scale ranging from 1 to 4	Sum of average scores of seven components	Components: subjective sleep quality, sleep latency, sleep duration, habitual sleep efficiency, sleep disturbances, use of medication, daytime dysfunction	0–21	Worse, with scores ≥ 6 considered poor sleep quality

^*^Using a factor analysis with an orthogonal varimax rotation, preliminary BCOPS data resulted in three factors. Active coping is comprised of active coping, planning, positive reframing and acceptance. Passive coping is comprised of self-distraction, denial, substance abuse, behavioral disengagement, venting and self-blame. Support seeking is comprised of instrumental support and emotional support

COPE measured how police officers cope with stress in their lives (Carver 1997). Three summary scales were derived from a factor analysis presented in a previous study and include active coping, passive coping, and support seeking (Andrew et al. 2013). The Hardiness Scale (Bartone 1985) and Connor and Davidson (2003), measured resiliency traits among officers (Bartone 1985). The Social Provisions Scale measured social support from relationships with family members, co-workers and community members (Table 1) (Cutrona 1987).

### Physical health outcomes

*Anthropometric measurements*. Anthropometric measurements (body mass index (BMI), abdominal height, waist circumference) and blood pressure (BP) were obtained by trained technicians. Resting heart rate (beats/minute) at supine position was measured three times and was averaged. Details on measurement protocols are presented elsewhere (Gu et al. 2012).

*Metabolic syndrome*. Metabolic Syndrome (MetSyn) is defined according to the criteria described by the Third Report of the National Cholesterol Education Program Adults Treatment Panel as the presence of three or more of the following five components: (1) Waist circumference (≥ 102 cm in men and ≥ 88 cm in women); (2) Systolic BP (≥ 130 mmHg or diastolic BP ≥ 85 mmHg or reported physician-diagnosed hypertension and antihypertension treatment); (3) Triglyceride (≥ 150 mg/dL or reported treatment with nicotinic acid or fibrates); (4) High density lipoprotein (HDL) cholesterol (< 40 mg/dL in men and < 50 mg/dL in women or reported treatment with nicotinic acid or fibrates); (5) Fasting glucose (≥ 100 mg/dL or reported treatment for diabetes) (Grundy et al. 2005).

*Laboratory tests*. Fasting was required for at least 12 h before blood specimen collection. Serum was removed after centrifugation and aliquots were frozen at -80º C. The tests included triglycerides, high-density lipoprotein (HDL) cholesterol, low-density lipoprotein (LDL) cholesterol, total cholesterol, glucose, C-reactive protein (CRP), leptin, white blood cell (WBC) count, hemoglobin AIC (%), and insulin. All tests were performed on serum (except for WBC counts which was performed on whole blood) using standard procedures. Details of all tests can be found in previously published articles (Charles et al.; 2015; Charles et al. 2011; McCanlies et al. 2011; Wirth et al. 2017).

*Brachial artery reactivity (BAR)*. BAR, a marker of subclinical CVD, is the dilation of the brachial artery after flow-mediated dilation (FMD) involving occlusion using a blood pressure cuff. FMD is a noninvasive method to quantify endothelial function. Lower FMD levels indicate endothelial dysfunction and early-stage atherosclerosis. Additional details regarding measurement of brachial artery FMD can be found elsewhere (Joseph et al. 2009).

*Carotid intima media thickness (IMT)*. The carotid IMT is a noninvasive method to identify atherosclerosis, a risk factor for CVD. Higher values suggest worsening atherosclerosis. Details of the assessment of carotid IMT have been previously described (Joseph et al. 2009).

A standardized B-mode ultrasound protocol was adopted from the Center for Medical Ultrasound at Wake Forest University, NC. B-mode ultrasound (Biosound Esaote, Indianapolis, IN) examinations were performed with a nominal center transducer frequency of 7.5–10 MHz. Mean common carotid IMT was derived as the average of the common carotid IMT measured at 12 sites in the right and left common carotid artery. Mean maximum IMT was the average of the maximum IMT measured at 12 sites on both the right and left sides of the neck and in the far and near walls of the common carotid, bifurcation, and internal carotid artery.

### Other outcomes

*Sleep*. The Pittsburgh sleep quality index (PSQI) questionnaire assessed sleep quality and quantity (Buysse et al. 1989) Nineteen self-rated questions assessed sleep quality related factors from the previous month (Table 1).

### Statistical analysis

We estimated the changes in demographic characteristics, occupational factors, and health outcomes across two time points and tested whether the changes were statistically significant: a 7-year change (baseline to 1st follow-up examination) and a 12-year change (baseline to 2nd follow-up examination). For continuous outcomes, the mean change between the two time points and the associated 95% confidence interval (CI) for the mean change were computed using the MIXED procedure in SAS (PROC MIXED). For binary outcomes, the change in prevalence between the two time points and the associated 95% CI was estimated using the GENMOD procedure in SAS (PROC GENMOD), with binomial distribution and the identity LINK function. For categorical outcomes (ordinal or nominal), we created a binary variable for each level and change in prevalence of each level and the associated 95% CI were estimated. In all three cases, the correlation of the two measurements within the same subject was accounted for by specifying the compound symmetry variance–covariance model. A change was considered statistically significant if the 95% CI did not include zero. All statistical analyses were performed using the SAS software version 9.4 (SAS Institute, Inc., Cary, NC).

## Results

Changes in selected demographic, lifestyle factors, and general health across two periods are presented in Table 2. The percentage of officers who were current smokers significantly decreased between the baseline and 1st follow-up examination by 5.9% (95% CI: − 9.4, − 2.5) and by 10.0% (− 15.0, − 5.1) between the baseline and the 2nd follow-up examination (both p < 0.001). The general health reported by officers somewhat worsened from baseline to the two time periods. Between the baseline and both the 1st and 2nd follow-up examinations, the percentage of officers who reported excellent/very good health significantly decreased by 11.8% (− 17.8, − 5.9) and 17.3% (− 24.2, − 10.4), respectively (both p < 0.0001). The percentage of officers reporting fair/poor health significantly increased by 5.1% (1.2, 8.9) between the baseline and 1st follow-up examination (p = 0.010) and increased but to a lesser extent between the baseline and 2nd follow-up examination by 4.2% (− 0.1, 8.5), p = 0.057. The percentage of officers who reported getting a routine physical exam once every year significantly increased between the baseline and both the 1st and 2nd follow-up examinations (both p < 0.0001).

**Table 2:** Comparisons of demographic characteristics of BCOPS study participants examined at baseline and subsequent follow-up examinations

Characteristics	Change from baseline to first follow-up (n = 276) Median time span = 7 years				Change from baseline to second follow-up (n = 191) Median time span = 12 years			
	Baseline	1st follow-up exam	Difference	p-value	Baseline	2nd follow-up exam	Difference	p-value
	Mean or % (95% CI)	Mean or % (95% CI)	Mean or % (95% CI)		Mean or % (95% CI)	Mean or % (95% CI)	Mean or % (95% CI)	
Age (years)	41.3 (40.3, 42.2)	48.2 (47.3, 49.2)	7.0 (6.9, 7.1)	< 0.0001	40.0 (38.9, 41.2)	51.9 (50.7, 53.0)	11.8 (11.6, 12.0)	< 0.0001
Years of service	14.5 (13.6, 16.5)	20.9 (20.0, 21.9)	6.4 (6.2, 6.6)	< 0.0001	13.1 (12.0, 14.2)	23.1 (22.0, 24.1)	10.0 (9.5, 10.4)	< 0.0001
Gender (male)	71.7 (66.4, 77.1)	71.7 (66.4, 77.1)	n/a	n/a	74.4 (68.2, 80.5)	74.4 (68.2, 80.5)	n/a	n/a
*Race/ethnicity*								
White	79.0 (74.2, 83.8)	79.0 (74.2, 83.8)	n/a	n/a	82.2 (76.8, 87.6)	82.2 (76.8, 87.6)	n/a	n/a
Black	18.5 (13.9, 23.1)	18.5 (13.9, 23.1)	n/a	n/a	16.8 (11.5, 22.1)	16.8 (11.5, 22.1)	n/a	n/a
Hispanic	2.5 (0.7, 4.4)	2.5 (0.7, 4.4)	n/a	n/a	0.1 (0.0, 2.5)	0.1 (0.0, 2.5)	n/a	n/a
*Marital Status*								
Single	13.1 (9.1, 17.1)	9.7 (6.2, 13.2)	− 3.4 (− 6.0, − 0.8)	0.009	13.6 (8.7, 18.5)	9.7 (5.6, 13.9)	-3.9 (-7.6, -0.2)	0.040
Married	73.4 (68.3, 78.7)	70.3 (64.9, 75.7)	− 3.1 (− 8.2, 1.9)	0.221	74.8 (68.7, 81.0)	70.3 (63.9, 76.8)	-4.5 (-11.1, 2.0)	0.177
Divorced	13.4 (9.4, 17.5)	20.0 (15.3, 24.8)	6.6 (2.1, 11.1)	0.004	11.5 (7.0, 16.0)	20.0 (14.3, 25.7)	8.5 (2.4, 14.5)	0.006
*Education*								
≤ 12 yrs	8.0 (4.8, 11.2)	8.3 (5.1, 11.6)	0.3 (− 2.4, 3.1)	0.814	7.3 (3.6, 11.0)	5.2 (2.1, 8.4)	-2.1 (-5.0, 0.8)	0.155
< 4 yrs college	54.7 (48.8, 60.6)	51.4 (45.6, 57.4)	− 3.3 (− 6.7, 0.2)	0.061	53.9 (46.9, 61.0)	52.9 (45.8, 60.0)	-1.0 (-4.8, 2.8)	0.593
4 + yrs college	37.3 (31.6, 43.0)	40.2 (34.4, 46.0)	2.9 (0.9, 4.9)	0.004	38.8 (31.8, 45.7)	41.9 (34.9, 48.9)	3.1 (0.7, 5.6)	0.013
*Smoking status*								
Current	15.7 (11.4, 20.0)	9.8 (6.3, 13.3)	− 5.9 (− 9.4, − 2.5)	0.001	15.8 (10.6, 21.0)	5.8 (2.5, 9.1)	-10.0 (-15.0, -5.1)	< 0.0001
Former	24.0 (19.0, 29.1)	30.9 (25.5, 36.4)	6.9 (3.4, 10.4)	< 0.0001	21.0 (15.2, 26.8)	30.4 (23.9, 36.9)	9.4 (4.1, 14.7)	0.001
Never	60.4 (54.6, 66.2)	59.3 (53.5, 65.1)	− 1.1 (− 2.3, 0.1)	0.084	63.4 (56.5, 70.2)	63.9 (57.1, 70.7)	0.5 (-1.8, 2.8)	0.649
*Rank*								
Police officers	70.9 (65.6, 76.3)	55.0 (49.1, 60.9)	− 15.9 (− 20.3, − 11.6)	< 0.0001	74.1 (67.9, 80.4)	43.0 (36.0, 50.1)	-31.1 (-37.8, 24.5)	< 0.0001
Serg/Lieut/Capt	16.9 (12.5, 21.3)	20.4 (15.6, 25.1)	3.5 (0.5, 6.5)	0.023	15.8 (10.5, 21.0)	28.0 (21.6, 34.4)	12.2 (6.9, 17.7)	< 0.0001
Detective	10.3 (6.7, 14.0)	20.7 (15.9, 25.5)	10.4 (6.7, 14.1)	< 0.0001	9.1 (5.0, 13.2)	25.9 (19.7, 32.2)	16.8 (11.5, 22.2)	< 0.0001
Others	1.7 (0.2, 3.2)	3.9 (1.6, 6.2)	2.2 (0.5, 4.0)	0.013	0.5 (0.0, 1.6)	2.7 (0.4, 5.0)	2.1 (-0.4, 4.6)	0.097
*General health*								
Excellent/V. good	55.7 (49.8, 61.6)	43.9 (38.0, 49.7)	-11.8 (-17.8, -5.9)	< 0.0001	59.7 (52.7, 66.6)	42.4 (35.4, 49.4)	-17.3 (-24.2, -10.4)	< 0.0001
Good	38.6 (32.8, 44.3)	45.3 (39.4, 51.2)	6.7 (0.0, 13.4)	0.049	36.7 (29.8, 43.5)	49.8 (42.7, 56.8)	13.1 (5.0, 21.2)	0.002
Fair/Poor	5.8 (3.0, 8.6)	10.9 (7.2, 14.5)	5.1 (1.2, 8.9)	0.010	3.7 (1.0, 6.3)	7.9 (4.0, 11.7)	4.2 (-0.1, 8.5)	0.057
*Routine phys. exam*								
Once/year	55.3 (49.4, 61.2)	68.9 (63.4, 74.3)	13.6 (7.1, 20.0)	< 0.0001	53.9 (46.9, 61.0)	78.5 (72.7, 84.4)	24.6 (16.8, 32.4)	< 0.0001
Once/5 years	26.0 (20.8, 31.2)	20.3 (15.6, 25.0)	− 5.7 (− 12.1, 0.6)	0.076	27.7 (21.4, 34.1)	14.1 (9.2, 19.1)	-13.6 (-21.1, -6.2)	0.001
< once/5 years	18.6 (14.0, 23.2)	10.9 (7.2, 14.5)	− 7.7 (− 12.6, − 2.8)	0.002	18.3 (12.8, 23.8)	7.3 (3.6, 11.0)	-11.0 (-17.0, -5.0)	0.001
Alcohol drinks/week	5.44 (4.34, 6.55)	5.38 (4.28, 6.49)	-0.06 (-1.13, 1.01)	0.915	4.63 (3.46, 5.79)	5.19 (4.03, 6.36)	0.56 (-0.75, 1.88)	0.398

Changes in occupational and psychosocial factors across the examination periods are presented in Table 3. The number of overtime hours per week significantly increased from baseline to both the 1st and 2nd follow-up examinations (p < 0.001). Changes in PTSD and anxiety scores were not significantly different across the time periods. Perceived stress decreased significantly over both time periods (p < 0.01). There was a slight increase in mean depressive symptoms from the baseline to the 1st follow-up examination (7.7 to 8.6, p = 0.031) but the change was not significant at the 2nd follow-up (7.3 to 6.6) (p = 0.198). The mean hostility and impact of event scores decreased significantly over the 12-year period (16.8 to 15.6, p = 0.016 and 11.7 to 8.7, p = 0.002, respectively). Police-specific work stress, as measured by the Spielberger Stress Scale significantly increased from baseline to the 2nd follow up examination [3.4 (1.2, 5.6)]; p = 0.003) including stress scores for physical/psychological danger which increased by 1.4 (p = 0.006) and lack of support which increased by 1.2 (p < 0.001).

**Table 3:** Comparisons of work related, psychosocial and protective factors of BCOPS study participants examined at baseline and subsequent follow-up examinations

Variables	Change from baseline to first follow-up (n = 276) Median time span = 7 years				Change from baseline to second follow-up (n = 191) Median time span = 12 years			
	Baseline	1st follow-up exam	Difference	p-value	Baseline	2nd follow-up exam	Difference	p-value
	Mean or % (95% CI)	Mean or % (95% CI)	Mean or % (95% CI)		Mean or % (95% CI)	Mean or % (95% CI)	Mean or % (95% CI)	
*Work-related factors*								
Regular shift hours/week	39.4 (38.9, 39.9)	40.2 (39.6, 40.7)	0.78 (0.15 1.4)	0.016	39.4 (38.8, 40.0)	41.5 (40.8, 42.1)	2.1 (1.2, 2.9)	< 0.001
Overtime/extended hours/week	3.3 (2.4, 4.1)	8.7 (7.9, 9.6)	5.5 (4.5, 6.4)	< 0.001	2.9 (1.9, 4.0)	11.1 (10.1, 12.1)	8.2 (7.0, 9.4)	< 0.001
Second job hours/week	12.0 (10.7, 13.4)	12.5 (11.1, 13.9)	0.45 (− 0.99, 1.9)	0.533	12.1 (10.2, 13.9)	13.4 (11.6, 15.2)	1.3 (− 0.97, 3.6)	0.248
*Shiftwork*								
Day	42.2 (36.2, 48.2)	47.3 (41.4, 53.2)	5.1 (1.1, 9.0)	0.013	36.3 (29.3, 43.4)	50.3 (43.2, 57.4)	14.0 (8.1, 19.8)	< 0.001
Afternoon	33.5 (27.8, 39.2)	33.1 (27.5, 38.7)	− 0.37 (− 4.3, 3.6)	0.855	32.4 (25.6, 39.3)	31.9 (25.3, 38.6)	− 0.5 (− 6.6, 5.7)	0.882
Night	24.3 (19.2, 29.5)	19.6 (14.9, 24.3)	− 4.7 (− 8.1, -1.3)	0.006	31.3 (24.5, 38.1)	17.8 (12.4, 23.2)	− 13.5 (− 18.8, − 8.2)	< 0.001
*Psychosocial factors*								
Perceived Stress	20.0 (19.1, 20.9)	18.6 (17.7, 19.5)	− 1.4 (− 2.3, − 0.50)	0.002	19.5 (18.4, 20.5)	17.2 (16.2, 18.3)	− 2.2 (− 3.3, − 1.2)	< 0.001
CES-D score	7.7 (6.9, 8.5)	8.6 (7.8, 9.4)	0.94 (0.09, 1.8)	0.031	7.3 (6.4, 8.2)	6.6 (5.7, 7.5)	-0.64 (-1.6, 0.34)	0.198
PTSD score	25.8 (24.8, 26.9)	26.4 (25.4, 27.4)	0.54 (− 0.52, 1.6)	0.314	25.5 (24.3, 26.8)	25.5 (24.3, 26.6)	− 0.07 (− 1.4, 1.3)	0.919
Beck Anxiety score	6.1 (5.3, 6.8)	5.9 (5.1, 6.7)	− 0.17 (− 0.92, 0.57)	0.647	6.0 (5.0, 6.9)	5.9 (4.9, 6.9)	− 0.08 (− 1.2, 1.0)	0.888
Beck Hopelessness score	2.0 (1.7, 2.3)	2.1 (1.8, 2.4)	0.05 (− 0.27, 0.37)	0.769	1.9 (1.6, 2.2)	1.5 (1.2, 1.8)	− 0.35 (− 0.68, − 0.03)	0.031
Cook-Medley Hostility score	17.3 (16.3, 18.3)	17.1 (16.1, 18.0)	− 0.25 (− 1.0, 0.53)	0.528	16.8 (15.6, 18.0)	15.6 (14.6, 16.7)	− 1.2 (− 2.1, -0.23)	0.016
Impact of Events score	11.8 (10.2, 13,3)	11.9 (10.3, 13.5)	0.15 (− 1.5, 1.8)	0.861	11.7 (10.0, 13.4)	8.7 (7.0, 10.3)	− 3.0 (− 4.9, -1.1)	0.002
*Spielberger Police Stress Score (raw)	23.4 (21.9, 24.9)	22.1 (20.6, 23.5)	− 1.34 (− 3.0, 0.31)	0.110	23.0 (21.2, 24.9)	26.4 (24.3, 28.4)	3.4 (1.2, 5.6)	0.003
Admin./Professional Pressure	7.8 (7.3, 8.4)	7.3 (6.8, 7.9)	− 0.51 (− 1.1, 0.06)	0.081	7.7 (7.0, 8.3)	8.4 (7.7, 9.1)	0.69 (− 0.08, 1.5)	0.078
Physical/Psychological danger	10.7 (10.0, 11.4)	9.9 (9.2, 10.6)	− 0.81 (− 1.6, − 0.03)	0.041	10.6 (9.8, 11.5)	12.1 (11.1, 13.0)	1.4 (0.42, 2.5)	0.006
Lack of support	4.8 (4.5, 5.2)	4.8 (4.5, 5.2)	− 0.03 (− 0.42, 0.37)	0.887	4.7 (4.2, 5.1)	5.9 (5.4, 6.4)	1.2 (0.69, 1.8)	< 0.001
*Protective factors*								
*Coping*								
Active	4.4 (4.3, 4.5)	4.4 (4.3, 4.6)	0.04 (− 0.11, 0.20)	0.576	4.4 (4.2, 4.6)	4.6 (4.4, 4.8)	0.18 (− 0.03, 0.39)	0.101
Passive	1.7 (1.6, 1.8)	1.6 (1.5, 1.8)	− 0.10 (− 0.18, − 0.01)	0.030	1.7 (1.6, 1.8)	1.4 (1.3, 1.5)	− 0.32 (− 0.42, − 0.22)	< 0.001
Support	3.5 (3.3, 3.6)	3.5 (3.3, 3.6)	0.01 (− 0.15, 0.17)	0.901	3.6 (3.4, 3.8)	3.6 (3.4, 3.8)	0.004 (− 0.20, 0.21)	0.970
*Hardiness*								
Commitment	10.3 (10.0, 10.5)	9.8 (9.6, 10.1)	− 0.42 (− 0.67, − 0.16)	0.002	10.4 (10.1, 10.7)	10.1 (9.8, 10.4)	− 0.29 (− 0.58, 0.01)	0.056
Control	9.9 (9.6, 10.1)	9.6 (9.4, 9.9)	− 0.24 (− 0.46, − 0.01)	0.039	10.0 (9.6, 10.3)	9.9 (9.6, 10.2)	− 0.03 (− 0.33, 0.27)	0.850
Challenge	8.4 (8.1, 8.7)	8.6 (8.3, 9.0)	0.27 (− 0.04, 0.58)	0.086	8.2 (7.9, 8.6)	8.6 (8.3, 9.0)	0.41 (0.03, 0.79)	0.035
Social Support	84.1 (83.0, 85.2)	83.7 (82.6, 84.8)	− 0.39 (− 1.4, 0.59)	0.439	84.5 (83.2, 85.8)	85.1 (83.8, 86.3)	0.56 (− 0.74, 1.9)	0.395

^*^Spielberger variables were scaled (divided by 100)

The mean scores for active and support coping did not change significantly across either of the time periods but passive coping scores decreased significantly over the 7- and 12-year periods [− 0.10 (− 0.18, − 0.01) and − 0.32 (− 0.42, − 0.22), respectively] (Table 3). Mean hardiness component scores for commitment and control decreased from baseline to the 1st follow-up but stayed relatively unchanged from baseline to the 2nd follow up examination (p = 0.002 and p = 0.039, respectively). Social support did not significantly change over either time periods.

Table 4 presents changes in physical health outcomes across the examination periods. The prevalence of Met Syn significantly increased by 10.7% (5.3–16.0) from the baseline examination to the 1st follow-up examination and by 7.4% (0.1–14.0) from the baseline to the 2nd follow-up exam. Of the five components of Met Syn, only abdominal obesity and glucose intolerance significantly increased over both time periods.

**Table 4:** Comparisons of health outcomes of BCOPS study participants examined at baseline and subsequent follow-up examinations

Variables	Change from baseline to first follow-up (n = 276) Median time span = 7 years				Change from baseline to second follow-up (n = 191) Median time span = 12 years			
	Baseline	1st follow-up exam	Difference	p-value	Baseline	2nd follow-up exam	Difference	p-value
	Mean or % (95% CI)	Mean or % (95% CI)	Mean or % (95% CI)		Mean or % (95% CI)	Mean or % (95% CI)	Mean or % (95% CI)	
*Metabolic syndrome factors*								
Abdominal obesity (%)	30.5 (25.1, 36.0)	45.7 (39.8, 51.5)	15.1 (9.6, 20.7)	< 0.0001	27.0 (20.7, 33.4)	43.1 (35.9, 50.4)	16.1 (9.5, 22.6)	< 0.0001
Elevated blood pressure (%)	34.5 (28.9, 40.1)	38.8 (33.0, 44.5)	4.3 (− 0.1, 9.3)	0.092	28.3 (21.9, 34.7)	32.8 (25.9, 39.6)	4.48 (-1.65, 10.6)	0.152
Glucose intolerance (%)	18.2 (13.6, 22.7)	29.7 (24.3, 35.1)	11.6 (5.9, 17.2)	< 0.0001	15.2 (10.1, 20.3)	26.9 (20.4, 33.4)	11.7 (5.3, 18.1)	0.001
Low HDL cholesterol (%)	36.7 (31.0, 42.4)	33.3 (27.8, 38.9)	− 3.3 (− 8.4, 1.8)	0.200	35.6 (28.8, 42.4)	35.2 (28.3, 42.2)	-0.38 (-6.64, 5.88)	0.905
Elevated triglyceride (%)	26.5 (21.3, 31.7)	28.3 (23.0, 33.6)	1.8 (− 3.2, 6.8)	0.486	25.1 (19.0, 31.3)	30.3 (23.6, 37.0)	5.18 (-0.85, 11.2)	0.092
Metabolic syndrome (≥ 3 components) (%)	20.5 (15.7, 25.3)	31.2 (25.7, 36.6)	10.7 (5.3, 16.0)	0.0001	18.2 (12.7, 23.7)	25.6 (19.1, 32.0)	7.4 (0.1, 14.0)	0.028
No. metabolic syndrome components (0–5)	1.47 (1.30, 1.63)	1.76 (1.59, 1.92)	0.29 (0.16, 0.43)	< 0.0001	1.33 (1.13, 1.52)	1.70 (1.50, 1.90)	0.37 (0.22, 0.52)	< 0.0001
*Pittsburgh Sleep Quality Index (PSQI)*								
Hours of actual sleep	6.10 (5.96, 6.25)	6.06 (5.92, 6.20)	− 0.04 (− 0.20, 0.12)	0.610	6.20 (6.03, 6.36)	6.06 (5.89, 6.23)	-0.13 (-0.33, 0.06)	0.175
PSQI global score (range: 0–21)	6.38 (5.96, 6.80)	8.38 (7.97, 8.79)	2.00 (1.62, 2.38)	< 0.0001	6.04 (5.56, 6.52)	8.16 (7.67, 8.66)	2.12 (1.68, 2.56)	< 0.0001
Poor sleep quality (%)	52.9 (46.8, 59.0)	80.8 (76.0, 85.5)	27.8 (21.2, 34.5)	< 0.0001	48.9 (41.7, 56.1)	77.1 (70.7, 83.4)	28.2 (20.0, 36.3)	< 0.0001
*Physical measurements*								
Body mass index (kg/m^2^)	28.8 (28.3, 29.4)	29.7 (29.2, 30.3)	0.88 (0.61, 1.16)	< 0.0001	28.5 (27.9, 29.2)	29.3 (28.7, 30.0)	0.79 (0.51, 1.07)	< 0.0001
Heart rate (beats/minute)	62.5 (61.5, 63.5)	64.5 (63.5, 65.5)	1.96 (0.98, 2.93)	< 0.0001	61.7 (60.6, 62.8)	63.6 (62.4, 64.7)	1.86 (0.69, 3.02)	0.002
Abdominal height (cm)	20.6 (20.2, 21.0)	21.7 (21.3, 22.1)	1.11 (0.82, 1.40)	< 0.0001	20.4 (20.0, 20.8)	21.4 (21.0, 21.9)	1.02 (0.70, 1.33)	< 0.0001
Waist circumference (cm)	93.1 (91.4, 94.8)	97.6 (96.0, 99.3)	4.54 (3.70, 5.39)	< 0.0001	92.7 (90.7, 94.6)	97.0 (95.1, 99.0)	4.35 (3.39, 5.31)	< 0.0001
Systolic blood pressure (mm/Hg)	120.5 (119.1, 121.8)	116.7 (115.4, 118.1)	− 3.75 (− 5.02, − 2.48)	< 0.0001	118.7 (117.2, 120.2)	115.6 (114.0, 117.2)	-3.09 (-4.60, -1.58)	< 0.0001
Diastolic blood pressure (mm/Hg)	76.8 (75.8, 77.9)	(78.3, 77.2, 79.3)	1.43 (0.35, 2.51)	0.010	75.9 (74.7, 77.2)	77.6 (76.4, 78.9)	1.71 (0.45, 2.96)	0.008
*Blood Measures*								
Triglyceride (mg/dL)	122.0 (111.3, 132.6)	121.5 (110.9, 132.0)	− 0.49 (− 10.42, 9.45)	0.923	115.3 (104.5, 126.0)	119.1 (108.2, 130.0)	3.80 (-5.49, 13.09)	0.421
HDL-Cholesterol (mg/dL)	47.8 (46.0, 49.5)	49.4 (47.7, 51.1)	1.63 (0.47, 2.80)	0.006	47.8 (45.7, 49.8)	49.1 (47.0, 51.2)	1.36 (0.02, 2.70)	0.046
LDL-Cholesterol (mg/dL)	126.8 (122.9, 130.8)	124.1 (120.1, 128.0)	− 2.79 (− 6.43, 0.86)	0.134	125.8 (121.1, 130.5)	121.0 (116.2, 125.8)	-4.85 (-9.49, -0.22)	0.040
Total-Cholesterol (mg/dL)	198.7 (194.3, 203.0)	197.4 (193.1, 201.7)	− 1.28 (− 5.33, 2.77)	0.533	196.5 (191.1, 201.8)	193.9 (188.5, 199.3)	-2.59 (-7.61, 2.43)	0.310
Glucose (mg/dL)	91.9 (89.8, 93.9)	96.8 (94.8, 98.8)	4.95 (2.60, 7.31)	< 0.0001	90.4 (88.8, 92.1)	93.9 (92.2, 95.6)	3.47 (2.03, 4.90)	< 0.0001
CRP (mg/dL)	3.13 (2.50, 3.75)	2.76 (2.14, 3.38)	− 0.37 (− 1.18, 0.45)	0.378	3.25 (2.40, 4.10)	2.32 (1.46, 3.19)	-0.93 (-2.08, 0.23)	0.116
Leptin (pg/mL)	13,098 (11,720, 14,475)	11,804 (10,435, 13,174)	− 1294 (− 2382, − 206)	0.020	12,047 (10,428, 13,667)	11,149 (9507, 12,790)	-899 (-2138, 340)	0.154
WBC count (× 10^3^ cells/µL)	5.82 (5.63, 6.01)	5.82 (5.63, 6.01)	− 0.00 (− 0.17, 0.16)	0.959	5.76 (5.54, 5.99)	5.76 (5.53, 5.99)	0.00 (-0.21, 0.21)	0.991
Hemoglobin A1C (%)	5.52 (5.46, 5.58)	5.48 (5.38, 5.51)	− 0.07 (− 0.14, − 0.01)	0.030	5.47 (5.41, 5.52)	5.36 (5.30, 5.42)	-0.11 (-0.15, -0.07)	< 0.0001
Insulin (µU/mL)	377.3 (324.9, 429.7)	434.8 (382.9, 486.6)	57.5 (− 6.14, 121.1)	0.076	344.0 (306.1, 381.8)	354.1 (315.7, 392.7)	10.2 (-26.1, 46.4)	0.580
*Measures of sub-clinical CVD*								
Percent change in brachial artery diameter	5.68 (5.33, 6.02)	3.97 (3.63, 4.31)	− 1.71 (− 2.15, − 1.26)	< 0.0001	6.08 (5.64, 6.53)	3.83 (3.38, 4.28)	-2.25 (-2.82, -1.68)	< 0.0001
Common carotid IMT (mm)	0.61 (0.60, 0.63)	0.67 (0.66, 0.69)	0.06 (0.05, 0.07)	< 0.0001	0.60 (0.59, 0.61)	0.66 (0.64, 0.67)	0.06 (0.05, 0.06)	< 0.0001
Maximum carotid IMT (mm)	0.88 (0.86, 0.90)	0.96 (0.94, 0.98)	0.08 (0.06, 0.10)	< 0.0001	0.86 (0.84, 0.88)	0.93 (0.90, 0.95)	0.07 (0.05, 0.09)	< 0.0001

Mean percent change in brachial artery diameter is defined as: mean of maximum (post-release value) minus mean of baseline (pre-inflation value) × 100

*CRP* C-reactive Protein, *WBC* White blood cell

The prevalence of poor sleep quality significantly increased over the 7- and 12-year time periods, 27.8% (21.2, 34.5) and 28.2% (20.0, 36.3), respectively (Table 4). Mean hours of sleep duration was approximately 6 h and did not significantly change over both time periods. In both the 7-year and 12-year follow up examinations, participants experienced a significant change in anthropometric measurements. BMI increased by 0.88 kg/m^2^ (0.61, 1.16) from baseline to the 1st follow-up examination and increased by 0.79 kg/m^2^ (0.51, 1.07) to the 2nd follow-up examination. Resting heart rate increased by 1.96 bpm (0.98, 2.93) and by 1.86 bpm (0.69, 3.02) over the two periods, respectively. Abdominal height and waist circumference also showed significant increases in the 7-year and 12-year follow-up examinations. Systolic BP decreased by 3.75 mm/Hg (-5.02, -2.48) and 3.09 mm/Hg (-4.60, -1.58) over the two periods, respectively. In contrast, diastolic BP increased by 1.43 mm/Hg (0.35, 2.51) and by 1.71 mm/Hg (0.45, 2.96) over the two periods, respectively.

HDL cholesterol increased by 1.63 mg/dL (0.47, 2.80) and by 1.36 mg/dL (0.02, 2.70) over the two periods, respectively. Glucose significantly increased over the two periods by 4.95 mg/dL (2.60, 7.31) and by 3.47 mg/dL (2.03, 4.90), respectively. LDL cholesterol significantly decreased from baseline to the 2nd follow-up examination, -4.85 mg/dL (-9.49, -0.22). We did not observe significant changes for triglycerides, total cholesterol, CRP, WBC count, and insulin levels from baseline to both follow-up examinations. The percent change in brachial diameter declined (worsened) from baseline to both follow-up examinations by 1.71% and 2.25%, respectively. Common and maximum carotid IMT increased (worsened) significantly from the baseline to both follow-up examinations.

## Discussion

This paper describes the changes in occupational stressors and various health outcomes for police officers over several years. Our findings show that the general health reported by officers worsened from baseline to subsequent examinations. Between the baseline and both the 1st and 2nd follow-up examinations, the percentage of officers who reported excellent/very good health declined significantly. It was noted that the percentage of officers who reported getting either a routine physical exam once every five years or less than once every five years significantly decreased over the 12-year period (i.e., baseline to second follow-up examination). These results are not unusual. Longitudinal studies indicate that physical fitness declines across time, particularly in individuals who do not remain physically active (Tittlback et al. 2005; Kozakai et al. 2020). Among officers, physical performance decreased by 10–32% over 16 years (Lagestad et al. 2014). Adults who remain physically active experience less decline across time than those who lack physical activity (Preuss et al. 2012). Future research could evaluate whether officers are offered incentives and given sufficient time to exercise.

Overall stress levels, as assessed by the Spielberger Police Stress Score instrument, significantly increased over a period of 12 years. In a study comparing stress levels in Swedish and Norwegian officers across time, the authors found that the Swedish officers did not have higher levels of stress, while the Norwegian officers did (Padyab et al. 2023). These differences may be the result of factors such as organizational stress or strained community relations (Saunders et al. 2019; Scott 2004). However, over that same 12-year period, our findings showed that feelings of hopelessness and hostility significantly decreased. Regarding protective factors for stress, active coping and support remained constant over time. Passive coping decreased significantly suggesting that officers might be using more effective strategies for coping with stress. More effective coping strategies include planning, seeking support or acceptance (Acquadro et al. 2015; Lazarus 1993) Also, problem focused coping is a positive strategy and includes interpersonal efforts to alter the stressful situation as well as efforts to solve problems related to stressors (Folkman & Lazarus 1986). Two elements of hardiness (a measure of resiliency), commitment and control, declined significantly over the first seven years but did not change substantially over the 12-year period. In contrast, the third element of hardiness, challenge, significantly increased over 12 years. Levels of social support remained somewhat constant over the examination periods. Several aspects of subclinical CVD appeared to increase over time. Brachial artery reactivity, a measure of artery health, significantly decreased over time suggesting poorer artery health and flexibility.

We observed that carotid IMT increased over time, suggesting a buildup of plaque. The prevalence of the MetSyn increased over the 12 years. Of the five MetSyn components, abdominal obesity and glucose intolerance significantly increased over both time periods. Hypertension and elevated triglyceride levels increased slightly but not significantly over both time periods while low HDL cholesterol did not increase over time.

In a previous study comparing police with the general working population, a higher percentage of officers were obese (40.5% vs. 32.1%), had the metabolic syndrome (26.7% vs. 18.7%), and had higher mean serum total cholesterol levels (200.8 mg/dL vs. 193.2 mg/dL) (Hartley et al. 2011). Age may also be considered as factor in these increases among police as well as the general population. The prevalence of CVD has been shown to increase with age, in both men and women, including the prevalence of atherosclerosis, stroke, and myocardial infarction (North & Sinclair 2012).

Many police organizations have begun to place an increased emphasis on wellness and prevention efforts. The finding that the life expectancy of white male police officers was, on average, significantly lower than that of the U.S. population underscores the need for emphasis on fitness throughout the police career (O’Malley 2022).

A wellness plan developed specifically for law enforcement personnel has shown promising results (Nice 2017). Surveys are first issued to employees to ascertain their wellness interests, preferences and readiness to improve their health. The results of surveys are shared with Command Staff/decision-makers. Program promotional materials are customized and provided to the agency/department. Points are earned by participants in the program for successfully practicing four key healthy habits (exercise, nutrition, sleep and stress management) and/or losing weight. LEO-specific wellness tips, strategies and support are emailed every week. Individual and team high achievers are rewarded with certificates signed by the Sheriff/Chief or other incentives that the agency/department deems appropriate.

The BCOPS study is somewhat limited due to the investigation of one specific police department which would likely affect generalizability of our findings to other police departments that have different characteristics. However, the findings of the BCOPS study may help to add to the vast literature on cardiovascular research. Also, the subjective nature of the self-report questionnaire may result in some bias.

Despite these general limitations, there are several strengths of the BCOPS study. Use of more quantitative or objective physiologic measures of subclinical CVD, metabolic derangement, and response to stress is an advantage. To the best of our knowledge, this is the only prospective study on police officers that compares both occupational factors and health outcomes over a significant period. Most studies on police officers have utilized cross-sectional designs. The few longitudinal or comparative studies that we have identified investigated a limited number of health markers (e.g., blood lipid profiles and physical fitness levels) or occupational stressors over a few years (Lockie et al. 2022; Padyab et al. 2023; Hansen et al. 2022; Magnavita et al. 2018). The longitudinal design allowed the calculation of change in subclinical markers and will enable the evaluation of the temporal aspects of relationships regarding indicators of stress and disease outcomes that are not available in cross-sectional or retrospective designs. The availability of an established cohort that includes many complex lifestyle habits, psychosocial factors, biometric characteristics, and subclinical structural/functional parameters enabled us to successfully complete the study. These strengths suggest that the BCOPS study will help to contribute new scientific evidence regarding the impact of occupational stress on markers of subclinical cardiovascular and metabolic outcomes and provide new insights into the health consequences of stress. The knowledge gained may contribute to both police and other first responders as well as to public health implications.

## Conclusions

One may conclude from the BCOPS study that, among police officers, many occupational factors related to stress, mental health, and CVD outcomes increase over time. The findings in previously published BCOPS studies suggest that the physical and mental health of police officers are adversely affected by certain occupational exposures. An important topic of consideration in police health research is the evaluation of programs which best address reduction of disease and mental health outcomes. The wellness program mentioned earlier is just one example of a possible strategy to help mitigate stress and adverse health outcomes among police. More exploration is needed for intervention at both organizational and individual levels. It is highly recommended that future studies compare the changes in general health status of law enforcement officers with that of the general population, ideally matching on age and sex. The BCOPS study research team will continue to conduct further analyses on the data from the three examinations to disseminate additional results to police agencies and occupational investigators.

## Data Availability

The SAS data used to support the findings of this Buffalo Cardio-Metabolic Occupational Police Stress (BCOPS) study have not been made available because of the sensitive nature of the health-related outcomes and the potential for participant re-identification.

## Abbreviations

BCOPS: Buffalo Cardio-Metabolic Occupational Police Stress
BMI: Body mass index
BP: Blood pressure
CES-D: Center for epidemiological studies-depression scale
CI: Confidence interval
CRP: C-reactive protein
CVD: Cardiovascular disease
FMD: Flow-mediated dilation
HDL: High density lipoprotein
LDL: Low density lipoprotein
IMT: Intima media thickness
PSQI: Pittsburgh sleep quality index
PTSD: Post-traumatic stress disorder
